# Somatic and visceral effects of word valence, arousal and concreteness in a continuum lexical space

**DOI:** 10.1038/s41598-019-56382-2

**Published:** 2019-12-27

**Authors:** Alessandra Vergallito, Marco Alessandro Petilli, Luigi Cattaneo, Marco Marelli

**Affiliations:** 1 Department of Psychology, University of Milano-Bicocca, Milan, Italy; 2 Milan Center for Neuroscience (NeuroMi), Milan, Italy; 3 Center for Mind/Brain Sciences (CIMeC), University of Trento, Trento, Italy

**Keywords:** Language, Sensorimotor processing

## Abstract

Although affective and semantic word properties are known to independently influence our sensorimotor system, less is known about their interaction. We investigated this issue applying a data-driven mixed-effects regression approach, evaluating the impact of lexical-semantic properties on electrophysiological parameters, namely facial muscles activity (left corrugator supercilii, zygomaticus major, levator labii superioris) and heartbeat, during word processing. 500 Italian words were acoustically presented to 20 native-speakers, while electrophysiological signals were continuously recorded. Stimuli varied for affective properties, namely valence (the degree of word positivity), arousal (the amount of emotional activation brought by the word), and semantic ones, namely concreteness. Results showed that the three variables interacted in predicting both heartbeat and muscular activity. Specifically, valence influenced activation for lower levels of arousal. This pattern was further modulated by concreteness: the lower the word concreteness, the larger affective-variable impact. Taken together, our results provide evidence for bodily responses during word comprehension. Crucially, such responses were found not only for voluntary muscles, but also for the heartbeat, providing evidence to the idea of a common emotional motor system. The higher impact of affective properties for abstract words supports proposals suggesting that emotions play a central role in the grounding of abstract concepts.

## Introduction

In the first chapter of *Harry Potter and the Philosopher’s Stone*, Prof. Dumbledore is speaking with Prof. McGonagall in front of Privet Drive 4, asking her to call *Voldemort* using his proper name instead of the expression *‘You-Know-Who’*. Prof. McGonagall flinches hearing Voldemort’s name said out loud, but Dumbledore seems not to notice her reaction and continue exclaiming that there is no reason to be frightened of pronouncing Voldemort’s name^1^.

It is surprising how the simple word “*Voldemort*” conveys a complex pattern of emotions and bodily reactions in the unfortunate Professor McGonagall, which goes further beyond the processing of the term itself. Such effect can be explained by the idea of affective semantics, a complex lexical feature that strongly depends on the individual’s personal experience and is partly independent from the semantic connotation of words. Net of other variables, emotionally salient words are processed more accurately and faster^2,3^ and elicit different neurophysiological responses^4–8^ as compared to neutral ones.

As Professor McGonagall’s reaction to “Voldemort” clearly points out, psychological affective states are intimately bound to the sensorimotor system^9^. A vast amount of literature describes specific autonomic and somatic motor activity in healthy humans following exposure to affective stimuli or induction of affective states^10–13^, which is considered as innate according to some scholars^14^. Strengthening the boundary between affective states and sensorimotor system, compelling evidence suggested that this relationship is bidirectional: not only emotions are able to induce changes at the body level, but also sensorimotor changes contribute to the affective experience^15–17^.

Motor behaviour is mediated by two independent systems: the voluntary (somatic) motor system and an involuntary (*visceral)* motor system^11,18,19^. Effector-wise, this distinction is misleading. Affective behaviour is generally involuntary and is expressed as a complex pattern of mixed somatic and autonomic movements. For example, the affective state of rage engages skeletal muscles (in body postures, facial expression and vocalization), smooth muscle (pupil dilators and hair erectors) and endocrine glands (adrenal medullary secretion of catecholamine). This concept of two motor systems, voluntary and affective, that share the same effectors is well-documented in brain disorders. Focal brain lesions can affect voluntary motor control of, for example, facial muscles, while maintaining spontaneous facial mimicry, like smiling for a joke^20,21^. Holstege^22^ introduced the concept of *emotional motor system*, which runs parallel to the voluntary motor system.

The actual relation between psychological emotional states and affective motor patterns is the topic of a century and a half-old debate^23^ regarding whether emotional motor manifestations have a role in self-awareness of affective states or are simple consequences of higher-order processes. Despite the considerable attention drawn in the scientific community by the relations between emotions and the emotional motor system, the role of affective motor output in lexical processing remains unclear. Some studies have investigated the psychophysiological aspects of emotional lexical processing, using different paradigms and techniques, and providing heterogeneous results^24–31^.

Among affective variables, valence and arousal have been acknowledged as the most basic dimensions of emotional information^32^. Specifically, valence has been defined as the way an individual judges a situation and determines the polarity of the emotional activation, going from unpleasant (e.g. *massacre*) to pleasant (e.g. *kiss*). Arousal, instead, is defined as the degree of excitement or activation which an individual feels towards a given stimulus, going from calm (*sofa*) to exciting (*passion*)^33^. The two variables are known to entertain a typical U-shaped relationship^34,35^, with words rated as very positive or very negative being typically more arousing than neutral ones. Such relation has been documented in behavioural, electrophysiological and neuroimaging studies^6–8^.

The increasing interest in the *embodied cognition theory*, according to which semantic connotations are dependent on the sensorimotor representations that they imply^36–42^, drove researchers’ attention to how affective variables interact with semantic ones. Among the latter, concreteness is probably the most studied property and its importance in psycholinguistic and memory studies cannot be understated^34^. Concreteness can be considered as the degree to which a word referent is related to a perceptible entity, going from abstract (*wit*) to concrete (*pizza*), and positively influences a broad range of cognitive domains, from language processing^43^ to episodic, recognition memory^44,45^ and working memory^46^.

In the last decades behavioural, neuroimaging and electrophysiological studies investigated the interaction between emotionality and concreteness^30,47–58^, leading to different results and conclusions^59^. On one side, a conspicuous body of evidence suggested that affective variables may play a role in the acquisition^54^ and processing of abstract knowledge^49,50,60^. However, some previous electrophysiological and electromyographic studies^31,53^ do not fit with a similar interpretation, reporting valence effects even for concrete items. Künecke and colleagues^31^, for example, recorded facial muscular responses during a visual recognition task and found an increase of activity in the corrugator muscle for negative concrete stimuli, while no valence effect was observed for abstract words. In an ERPs study by Palazova *et al*.^53^, the EPN (i.e., a posterior negative component associated to emotional stimuli) recorded during a lexical decision task involving stimuli with different valence, emerged earlier for concrete as compared to abstract verbs.

A limitation of many of these studies, however, is that they employed experimental manipulations built on “extreme” examples of inherently continuous variables:^61,62^ there is not such a thing as an emotional vis-à-vis neutral word, or a positive vis-à-vis negative one: indeed, words may be affectively connoted to different degrees. This dichotomization practice raises well-known statistical concerns^63^, and the narrow focus of the corresponding experimental scenarios limits the generalizability of results. Moreover, as previously reported for word recognition studies^62,64^, researchers’ implicit knowledge can influence item selection, hence affecting the obtained results. A strong example in this sense comes from a previous study by McKoon and Ratcliff^65^, in which authors were able to predict results in a priming semantic task using their own intuitions.

We reasoned that stronger evidence supporting the embodiment of word meanings would come from recording automatic responses to words distributed along a continuum characterized by three different dimensions, namely valence, arousal and concreteness. A similar approach would be also useful to reduce the noise induced by individual experience in semantic processing. For example, if we tested Dumbledore and Bellatrix Lestrange as participants presenting the words *Voldemort* and *Death Eaters* it is highly possible that we won’t be able to produce coherent results. Indeed, their individual feelings about the word referents are considerably different. In line with this reasoning, using words covering a wide variable range is more likely to produce results that suffer less of inter-subjects’ variability.

Following these premises, in the present work, we adopted an experimental approach that avoids a-priori manipulations: electrophysiological responses were collected during passive listening of words, that is, as elicited directly by word properties with no need of dedicated experimental scenarios or manipulations. Differently from previous studies, responses were measured for both somatic and visceral activities, in order to investigate whether lexical processing impacts physiological responses at both autonomic and somatic level, thus providing further evidence to the idea of an underlying emotional motor system. In fact, we recorded electromyographic activity from three facial muscles, namely the left *corrugator supercilii* (from here on only *corrugator*), *zygomaticus major* (*zygomaticus)* and *levator labii superioris* (*levator labii*), and changes in the heart rate (HR). The choice of recorded muscles was aimed at maximizing the information obtained with the minimum number of recordings. The muscle pair zygomaticus/corrugator is canonically considered as exhaustive regarding the distinction between positive and negative emotional valence^19,66–69^ and more specifically distinguishes between happiness, anger and sadness. In addition, we recorded the levator labii muscle which is part of the mimic pattern of sadness, disgust and fear, so that all basic affective facial postures were detectable.

We adopted an exploratory approach: indeed, our hypothesis was that our variables of interest, namely affective and semantic variables, would have interacted, but we did not have strong predictions concerning the direction of such effect or the pattern of this interaction. In line with these premises, electrophysiological indexes were used as dependent variables and were separately submitted to a series of linear mixed-effects regressions, using a backward model selection procedure starting from a model containing the interaction between the three variables of theoretical interest, namely concreteness, valence and arousal, plus the effect of four control variables, namely word length, frequency, age of acquisition and number of orthographic neighbours. All predictors were operationalized as continuous variables, and random-effect intercepts were included in order to account for idiosyncratic effects of individual words.

## Results

A significant three-way interaction between concreteness, valence and arousal was found to influence both *corrugator* activity and HR (F (1,479.09) = 4.33, p = 0.038 and F (1,9670) = 4.725, p = 0.034, respectively).

**Figure 1:**
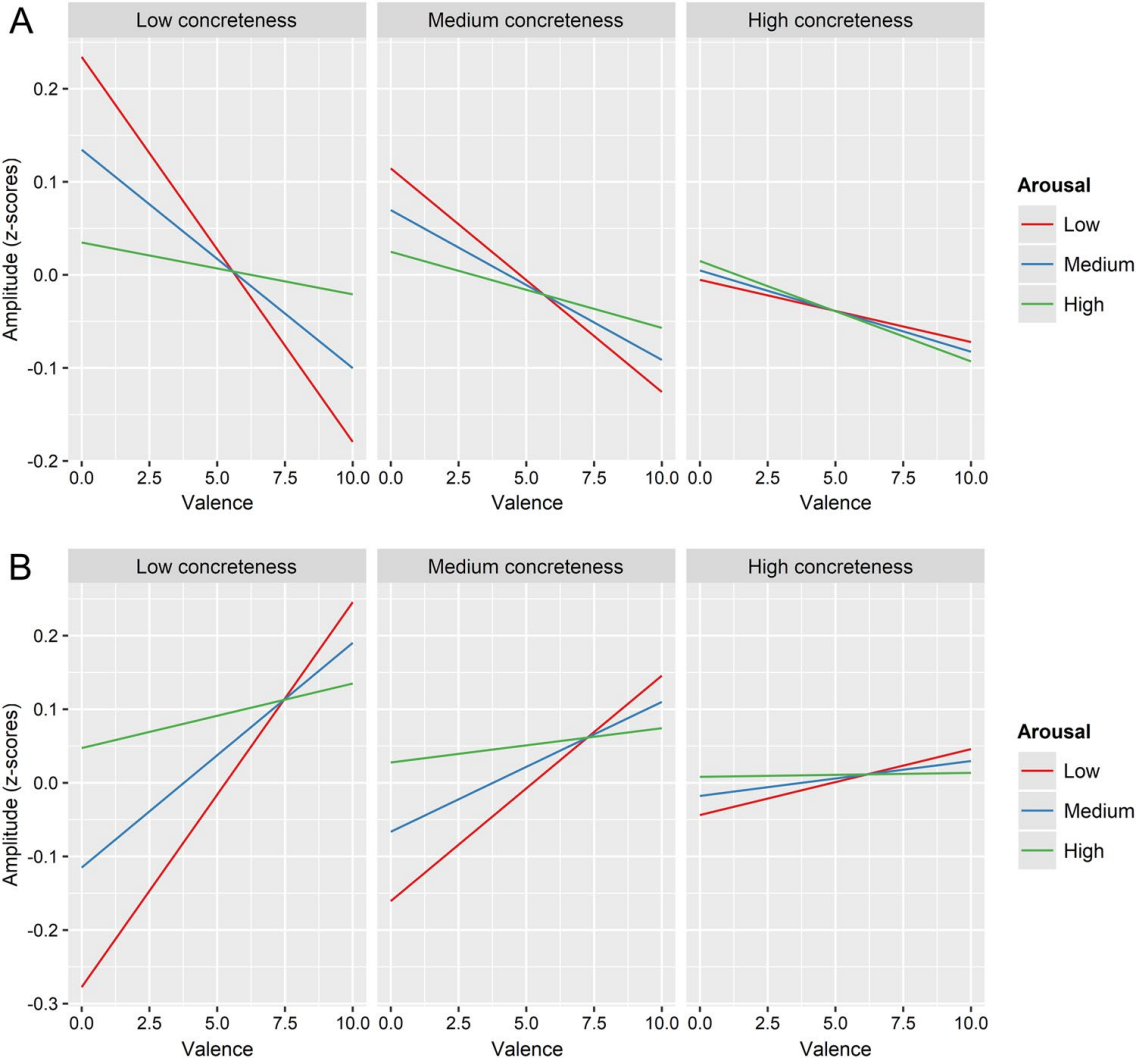
Interaction among valence, arousal and concreteness for corrugator and heart rate. The figure represents the three-way interaction among valence, arousal and concreteness on electrophysiological amplitude. Specifically, Panel (A) illustrates the interaction for corrugator amplitude, while Panel (B) highlights the interaction for HR changes. Note that analyses were conducted on continuous predictor variables; the categorical depiction of concreteness and arousal is merely used for the purpose of graphical representations.

Specifically, in the *corrugator* muscle EMG activity increases during the listening of more negative words, but mostly at lower levels of arousal (e.g. *bancarotta, bankrupt*) (see red lines in the upper panels of Fig. 1, and in particular the upper-left panel); in fact, the higher the arousal (e.g., *soffocare, suffocate*), the more the valence effect becomes smaller. In other words, the corrugator muscle shows polarity-specific activations, with stronger responses for negative vis-à-vis positive words, which is however more evident when the affective connotation of the word (in terms of arousal) is more subtle and tends to be substituted by a non-polarity-specific activation for very arousing words.

Valence-wise, an opposite pattern is observed for HR: heart activity tends to accelerate when more positive words were presented. However, also in this case, the effect was stronger at lower levels of arousal (e.g., *rilassato, relaxed*) (see red lines in the lower panels of Fig. 1, and specifically the lower-left panel). In other words, HR shows also polarity-specific activations, with stronger responses for positive vis-à-vis negative words, which is however more evident when the affective connotation of the word is more subtle (low-arousal words), and tends to be substituted by a non-polarity-specific activation for very arousing words (e.g. *passione, passion*).

Crucially, for both corrugator muscle and HR, the interaction between the two affective variables is modulated by the degree of concreteness of the presented word: the interaction is most evident for more abstract words (e.g., *prestigio, prestige*) (left panels of Fig. 1) and progressively decreases for words with higher levels of concreteness (e.g., *pizza, pizza*) (right panels of Fig. 1).

**Figure 2:**
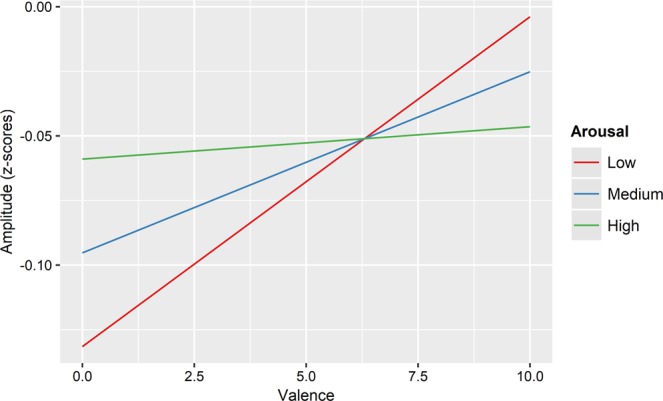
Interaction between valence and arousal on levator labii. The figure represents the two-way interaction between valence and arousal on levator labii EMG activity.

Statistical analysis of *levator labii* muscle responses showed a two-way interaction between valence and arousal (F (1, 509.13) = 4.35, p = 0.037). Indeed, similarly to what found for abstract words and the HR, the more positive the word, the larger the observed EMG activity. However, this polarity-specific response is more evident for low-arousal words (e.g., *casa*, *home*), while a more non-specific activation is found for high-arousal stimuli (e.g. *vittoria, victory*) (see Fig. 2).

In the zygomaticus muscle, only the effect of the orthographic neighbours covariate was significant (F(1,501.12) = 4.57, p = 0.033). Full statistical details of the four analyses are reported in the Supplementary Materials.

## Discussion

In the present work, we investigated the involvement of the somatic and visceral systems in lexical processing by measuring spontaneous electrophysiological activity in a continuum lexical space and evaluating the impact of intrinsic affective and semantic properties of words.

To this aim, twenty Italian native speakers took part in a two-session experiment, in which they passively listened to pre-recorded acoustic words, varying according to the valence, arousal and concreteness dimensions. For each participant, four electrophysiological indexes were continuously recorded: three measuring facial EMG activity, from corrugator, zygomaticus and levator labii muscles, and one measuring changes in HR.

The present results clearly indicate that both the visceral and the somatic systems are activated during word processing. Remarkably, this activation seems to be highly consistent, since a very similar pattern for valence and arousal is observed for two out of three facial muscles and HR. These results suggest that both the somatic and autonomic systems underlie similar processes with respect to affective semantics, with non-specific responses for very activating (i.e., high-arousal) words, and polarity-specific responses for words whose affective connotation is more nuanced. Facial and cardiac measures assess two completely different effector types (somatic and visceral). Therefore, the co-variance between the two measures indicates that, in physiological terms, we are indeed assessing a supra-modal affective system rather than a low-level output. The main variability in the response seems to be related to a channel-specific sensitivity to word polarity: when processing low-arousal items, some channels (*levator labii* muscle, HR) respond more to positive words, other channels (*corrugator* muscle) are more responsive to negative ones.

Such a pattern is only partially consistent with a previous proposal^70^, which interprets emotional word processing in terms of approach (low arousal and positive valence words) and avoidance (high arousal and negative words),and has received support in behavioural, neuroimaging (fMRI) and electrophysiological (ERPs) studies^6^. Indeed, in line with the model, in the present work HR and levator labii were more responsive to low arousing positive words; however, at the opposite, the corrugator activity was more responsive to low arousing negative stimuli, which is not consistent with the proposal. We can speculate that such a difference may be due to the general and a-specific activation induced by high-arousal words at a somatic and visceral level that may have masked the effect of valence. The motor “dominance” of high-arousing words can be explained from an evolutionary perspective. Indeed, affective processing relies upon a phylogenetic ancient system, directly finalized to survival. In this perspective, it is crucial that the motor system is activated in the presence of a salient stimulus, in order to prepare for a response (e.g. fight or flight). When the level of arousal induced by the presented stimulus decreases, only a polarity-specific response is traceable, since only channels specialized for specific emotional responses reach a certain activation threshold. In this case, in line with previous literature, we found corrugator activity increasing during more negative stimuli presentation^26,31,71^ and HR increasing with more positive valence words, which is compatible with a previous study by Ilves & Surakka^28^ in which a decreasing HR for negative words was reported. Surprisingly, we also found an effect for *levator labii* EMG activity, which increased for more positive valence words. Such result was unexpected; indeed, researchers rarely record this muscle, which response is usually related to the expression of disgust^72^.

Such affective-muscular specificity is likely based on a simulation process^73^, which triggers activity in the same brain regions and peripheral muscles involved in the execution of related emotional expressions^17,74–76^. For example, negative stimuli activate the *corrugator* muscle, which is used to approximate the eyebrows when frowning, while happy expressions have been found to increase EMG activity in the *levator labii*, which previous research suggested to be involved in forming a smile^77^.

However, the reported responses are not limited to affective aspects only: the observed emotional processing is crucially entangled with an impact of the semantic properties of the presented word, namely its degree of concreteness: the interaction between the affective variables is mostly observed for abstract words. These surprising results inform one of the most debated questions in modern psycholinguistics, namely how we do represent word meanings. According to *embodied semantics*, semantic connotations are dependent on the sensorimotor representations that they imply; this proposal is supported by several pieces of evidence from both experimental psychology and cognitive neuroscience^36–42^. However, while there is a general agreement that basic sensory or motor concepts are necessarily dependent on sensorimotor experience – you cannot give a meaning to the word “green” if you have never seen the colour green – there is a considerable discussion on the embodiment of abstract concepts^59^.

At first glance, our results may appear counterintuitive from an embodied perspective, which claims that word meanings are grounded in our sensorimotor states acquired during experiences with the word referents^78,79^. In fact, following these premises, one would expect stronger embodied effects for more concrete words, since only concrete objects are directly experienced in our everyday interactions with the environment. However, current approaches are trying to go beyond the concrete/abstract dichotomy. For example, the Affective Embodiment Account^2,49,50^ proposed a unified view of the Embodied Theory, which suggests that abstract and concrete concepts are both grounded in our experiences, although they are represented in different formats. Specifically, concrete concepts would be based on our perceptual and motor knowledge, while abstract concepts would rely on affective and emotional experiences. The idea that internal affective states may play a role in representing abstract words and concepts is not new^80^ and has been recently supported by both behavioural and neuroimaging evidence^81,82^. Barsalou and Wiemer-Hastings^83^ also suggested that abstract concepts and word meanings are grounded in internal mental and affective states, a perspective recently adopted by other researchers^84–87^. Our results are in line with this hypothesis: the electrophysiological responses that we recorded in our experiment, arising from bodily states controlled by the emotional motor system, are particularly involved in the affective processing of abstract words.

Our findings are not fully consistent with a previous EMG experiment by Künecke and colleagues^31^. These authors measured facial muscle activity during a visual word recognition task and found an increase in corrugator activity during the presentation of negative-valence words, but only with concrete items. However, our experiment presents a series of methodological differences with respect to Künecke *et al*.’s work. First, the proposed task was different (passive listening vs. visual lexical decision). Concerning this point, we are aware that auditory stimulation is not the most frequently used modality in this type of experiment. However, auditory presentation presents several advantages. It is the sensory channel that is hard-wired in our brain for linguistic communication, whereas written language is only an acquired skill. The auditory presentation allows passive exposure to the stimuli, which is a great advantage when it comes to recording facial EMG since covert reading is associated with motor activity such as saccades, head rotation and, most importantly, sub-vocal rehearsal in articulatory muscles. The main disadvantage is that the timing of access to the word semantics is less synchronized with auditory stimuli than with visual ones, but we addressed this point by time-locking the window of analyses to the end of each word stimulus.

Second, in our experimental design we set a longer inter-trial interval (9 seconds, compared to the 2 seconds of Künecke *et al*.), which is likely to deeply impact this kind of signals for two reasons: (i) in spite of their name, rapid facial reactions to affective stimuli occur in the time-range of hundreds to thousands of milliseconds, and allowing only 2 seconds between stimuli may produce considerable inter-stimulus interference; (ii) most importantly, affective, arousal and autonomic responses all show marked habituation to frequent stimuli^88–90^. In line with the authors, however, we did not find a significant effect over the zygomaticus activity, supporting the idea that this muscle is sensitive to the valence effect in pictures, sounds and facial expressions presentation^72,91^ but not in word processing^31,71^. Taken together, our results provide support to embodied cognition proposals. The use of continuous variables and spontaneous activity recording allows indeed to generalize the idea that language processing *automatically* activates the re-enactment of meaning-related body states, which can be observed with no need for dedicated experimental manipulations. Moreover, our data support the idea of an embodied grounding not limited to  concrete, but extended to abstract concepts, thus providing experimental evidence against the traditional dichotomy by showing a remarkable involvement of the affective system in the comprehension of abstract concepts.

## Methods

### Participants

20 healthy participants (10 males, *MAge* = 23.25, DS ± 3.1) took part in the study, which was run at the University of Milano-Bicocca. All participants were Italian native speakers and right-handed. They were naive about experimental procedures and aims of the study.

The study was approved by the ethical committee of the Department of Psychology of the University of Milano-Bicocca and was run according to the principles of the Declaration of Helsinki. Participants were invited to take part in the study through announcements on the Sona-System, an on-line platform used for participant recruitment (https://milano-bicocca.sona-systems.com), in exchange for course credits.

### Acoustic stimuli

Stimuli were acoustically presented through intra-aural headphones. 500 random words were taken from the Italian version of the ANEW dataset^92^. The obtained stimulus set included nouns (70.8%), adjectives (17.8%), verbs (5.8%), and words that could be considered either an adjective or a noun (5.6%).

**Figure 3:**
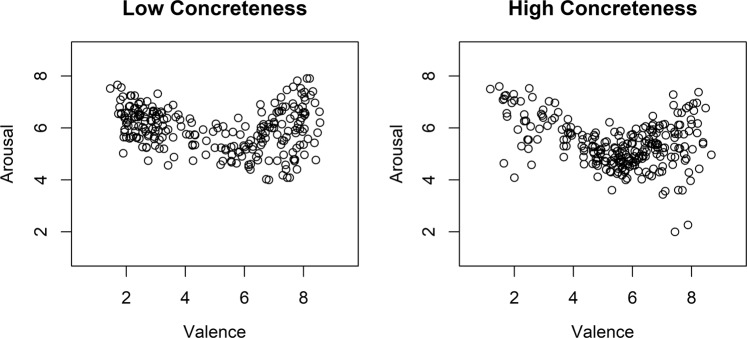
Distribution of affective variables in words with low and high concreteness. The figure represents valence (x-axis) and arousal (y-axis) distribution for low- and high-concreteness stimuli.

Concreteness, arousal, and valence norms were extracted from the Montefinese dataset and had been originally obtained through rating studies over 1084 participants. Previous works reported that abstract and concrete words are different in terms of affective ratings^2^. To address this potential concern, we explored the distribution of affective variables in our word sample and replicated the well-known U-shaped interaction^93^ for both low and high concrete items (defined via mean-splitting, see Fig. 3): in our item set affective variables are similarly distributed at different degrees of concreteness.

We converted each text word in speech format by using Balabolka, a Text-To-Speech freely available computer program (version 2.14, http://www.cross-plus-a.com/it/balabolka.htm), in this way stimuli were not associated with particular prosodic patterns. Words duration depended on Italian word length and had a range varying from 378 ms (‘RE’, *king*) to 1239 ms (‘DISINCANTATO’, *disenchanted*).

### Procedure

Participants were tested individually. Each one took part in two experimental sessions in two different days, at the same time of the day. Each session comprised 250 words. In the first session, participants received information about the experiment and gave their written informed consent prior to the experimental procedure.

Three pairs of electrodes were placed over the left corrugator, zygomaticus and levator labii muscles, following Fridlund and Cacioppo guidelines^94^, (for an overview of the anatomy of facial muscles see^95^). The ground electrode was placed at the midline, at the border of the hair line^72^. A pair of electrodes was placed on both arms, to record the heartbeat.

In order to avoid participants focusing their attention only on facial muscles, we added two fake electrodes on both ankles and told them we were interested in recording individual’s general psychophysiological state. They were asked to close their eyes, relax and focus on the meaning of the presented words. During the experiment, participants sat in a comfortable chair. A break was programmed every 83 trials, but participants could ask to stop any time they needed.

Each acoustic word was presented with an inter-stimulus interval of 9 seconds. Word order was randomized across participants. In order to maintain participants engaged, 10% of trials were followed by a question concerning the word meaning (e.g., “Did the previous word refer to an animal?”), and participants were instructed to respond by pressing the left (“yes”) or right (“no”) mouse key with their right index/medium finger.

The experiment was programmed using MATLAB 2017b (The MathWorks, Natick, Massachusetts, United States) and the Psychophysics Toolbox extension^96^.

### Electrophysiological recordings

Physiological parameters were recorded through 4-mm diameter surface Ag/AgCl filled with a Ten20 conductive paste (Weaver & Co., Aurora, CO, USA).

Electrophysiological activity was amplified 1000x with the Digitimer D360 amplifier (Digitimer Ltd., Welwyn Garden City, Hertfordshire, UK) sampled at 1000 Hz. The EMG signal was digitized using an analogue-digital converter (type 1401, Cambridge Electronic Design, Cambridge, UK) and recorded through Spike-2 software (Cambridge Electronic Design, Cambridge, UK) on a PC. Continuous electrophysiological recordings were collected for the two experimental sessions.

### Electrophysiological pre-processing

Data processing was performed in MatLab (The Mathworks, Natick, MA) using scripts based on EEGLAB 13.5.4 (sccn.ucsd.edu/eeglab), an open-source environment for processing electrophysiological data^97^.

The pre-processing pipeline for EMG data consisted of the following steps:

(1) The digitized EMG signals from facial muscles were filtered offline with a 30-highpass filter, and a 50-Hz notch filter was applied to remove line noise; (2) Continuous data were initially epoched from −500 to 7500 ms relative to the auditory stimulus onset and baseline corrected (−500 to 0 ms); (3) Epochs containing abnormal EMG activity were marked by using jointprob (epochs that deviated more than two standard deviations from the distribution mean) and rejkurt (kurtosis outside of two standard deviations of the mean kurtosis value) Matlab functions implemented in the EEGLAB toolbox^97,98^. Marked epochs were then visually inspected. Only epochs containing artefactual activity within 500 ms before the onset and 2000 ms after the offset of the stimuli were rejected. (4) The remaining epochs were rectified. (5) For each participant and session, epochs with baseline activity of more than 2 SD from the mean baseline amplitude across all trials were marked for rejection. (6) Overall, 4.6% of trials were removed from further analysis. (7) EMG signals were then time-locked to the end of each word. (8) EMG responses were defined as mean deviations of the first 1500 ms after the stimulus offset from the mean baseline activity (−2000 to 0 ms pre-stimulus activity). (9) Data were transformed in Z-scores.

**Figure 4:**
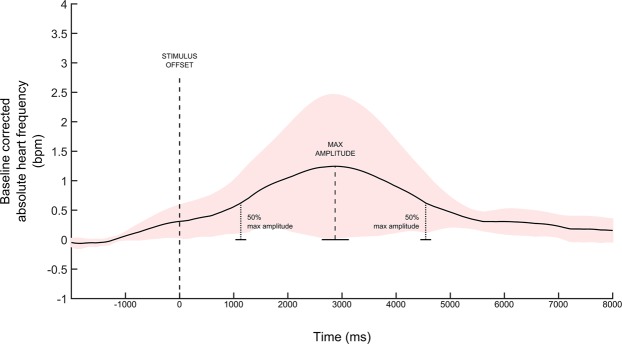
Heart rate changes over time. The figure represents the mean change in heart rate from 1000 ms before and 8000 ms after stimulus offset. The selected time-window corresponds to the two time-points at which the waveform passed the 50% of the maximum amplitude compared to the baseline.

The pre-processing pipeline adopted for the ECG data follows what is typically done in literature, namely considering the baseline-corrected instantaneous heart rate frequency in a variable window of maximum 6 seconds following the stimulus (e.g.^11,99,100^). Specifically, the pre-processing pipeline consisted of the following steps:

(1) The ECG signals were filtered offline with a 15-highpass and 20-lowpass filter^101^. R-wave peaks were detected automatically by means of ‘findpeaks’ Matlab function. (2) For each peak, the inter-beat interval (i.e., latency between each peak from the preceding one) was calculated. (3) Inter-beat interval deviating more than 3 scaled median absolute deviations from the median local heart rates within a sliding window of 10 points were removed (using MatLab function: “*isoutlier”*). (4) Instantaneous heart rate for each peak was then computed as the time unit (60 seconds / inter-beat interval). (5) Instantaneous heart rate was then estimated continuously for each time point of the ECG recording by fitting a cubic spline function over all estimated local heart rates. The estimation of continuous heart rate is beneficial for extracting the most informative time window from this signal, which is sparse and has a low sample rate (around a beat every 900 ms). (6) Continuous heart rate was then epoched from −2000 relative to stimulus onset to 6000 from stimulus offset. (7) Data averaged over all trials and participants highlighted a positive deflection of the heart rate at around 3000 ms after the stimulus offset (Fig. 1). Not having an *a-priori* hypothesis of the time window best-suited for the heart rate analysis, boundaries of the time-window of interest were defined as the two time-points at which the waveform passed the 50% of the maximum amplitude compared to the baseline (1130 ms – 4546 ms, see Fig. 4); ECG responses were defined as deviations of the mean heart rate in the selected window from the mean baseline (between −2000 and 0 ms relative to the onset of the stimulus^102^). Data were transformed in Z-scores.

### Statistical analysis

All participants had accuracy in catch trials above the 80% (mean of accuracy = 93.5, standard deviation = 3.5).

Analyses were performed in the statistical programming environment R^103^ using linear mixed-effects models (including non-linear interactions, as captured by tensor products in Generalized Additive Models^104^, did not result in improvements in terms of model fit) as statistical procedure^105^. EMG activity of the corrugator, zygomaticus, levator labii muscles and heart rate frequency were separately submitted to a series of linear mixed-effects regression using the lme4 package (version 1.1–17^106^); p-values were estimated by means of the lmerTest package (version 2.0–30^107^).

Concreteness, valence and arousal, namely the three variables of interest, were entered in each model as continuous fixed predictors. Log-transformed frequency (subtlex-it, http://crr.ugent.be/subtlex-it/), age of acquisition^108^, word length and the orthographical neighbors^92^ were entered in each model as control variables. Concerning the random structure, a by-item random intercept was included. At first, also by-subject and by-session components had been also considered but were then removed because they did not significantly improve model fit. After having fitted the full model, overly influential outliers were removed via model-criticism (2.5 SD of standardized residuals). With this procedure we eliminated 2.6% of datapoints in the corrugator analysis, 2.7% of datapoints in the levator labii analysis, 2.6% of datapoints in the zygomaticus analysis, and 2.5% of datapoints in the HR analysis.

Finally*, step* function (from the lmerTest package, version 2.0–30^107^) was applied as backward selection procedure to simplify each statistical model, thus eliminating non-significant fixed effects. Details of model selection are reported in the Supplementary Materials.

## Supplementary information

Table S1

Table S2

Table S3

Table S4

Table S5

Table S6

Table S7

Table S8

Readme

Supplementary Dataset 1

## Data Availability

The dataset generated during the current study is available as Supplementary materials and analyses or scripts are available from the corresponding author on reasonable request.
