# Supplementary material for: Somatic and visceral effects of word valence, arousal and concreteness in a continuum lexical space
Source: Sci Rep. 2019 Dec 27;9:20254. doi: 10.1038/s41598-019-56382-2 (PMC6934768; doi:10.1038/s41598-019-56382-2)
Supplement: Supplementary file 2 — Table S2 [file 41598_2019_56382_MOESM2_ESM.pdf]

Somatic and visceral effects of word valence, arousal and concreteness in a continuum lexical space

Alessandra Vergallito [1,2+]*, Marco Alessandro Petilli [1+], Luigi Cattaneo [3,4], Marco Marelli [1,2]

1 Department of Psychology, University of Milano-Bicocca

2 Milan Center for Neuroscience (NeuroMi),

3 Center for Mind/Brain Sciences (CIMeC), University of Trento

4 Department of Neuroscience, Biomedicine and Movement, University of Verona, Verona, Italy

+ AV and MAP equally contributed to the manuscript

*Corresponding author:

alessandra.vergallito@unimib.it,

Department of Psychology, University of Milano Bicocca,

Piazza Ateneo Nuovo, 1, 20126 Milano, Italy.

Model selection on the corrugator muscle

Parameter removed	*Degree of Freedom*	*Resulting AIC*	*Log likelihood*	*p*
Full model	14	22365	*-11168*	-
Orthographic neighbours	13	22363	*-11169*	*.7164*
Frequency	12	22362	*-11169*	*.5186*
Age of acquisition	11	22361	*-11169*	*.2668*
Length	10	22360	*-11170*	*.1936*
Concreteness : Valence : Arousal	9	22363	*-11172*	*.0364*

Table S2 summarizes degree of freedom, AIC, Log likelihood and p values of each model.

The first column ("parameter removed") indicates the removed parameter. Subsequent columns show the impact of the parameter removal in terms of model fit. Please note that models are in hierarchical level, starting from the full model and removing a parameter for each step.
