# Supplementary material for: Somatic and visceral effects of word valence, arousal and concreteness in a continuum lexical space
Source: Sci Rep. 2019 Dec 27;9:20254. doi: 10.1038/s41598-019-56382-2 (PMC6934768; doi:10.1038/s41598-019-56382-2)
Supplement: Supplementary file 3 — Table S3 [file 41598_2019_56382_MOESM3_ESM.pdf]

Somatic and visceral effects of word valence, arousal and concreteness in a continuum lexical space

Alessandra Vergallito [1,2+]*, Marco Alessandro Petilli [1+], Luigi Cattaneo [3,4], Marco Marelli [1,2]

1 Department of Psychology, University of Milano-Bicocca

2 Milan Center for Neuroscience (NeuroMi),

3 Center for Mind/Brain Sciences (CIMeC), University of Trento

4 Department of Neuroscience, Biomedicine and Movement, University of Verona, Verona, Italy

+ AV and MAP equally contributed to the manuscript

*Corresponding author:

alessandra.vergallito@unimib.it,

Department of Psychology, University of Milano Bicocca,

Piazza Ateneo Nuovo, 1, 20126 Milano, Italy.

Model selection on the levator labii superioris

Parameter	χ2	*p*	*Removal order*	*Estimate*	*t-value*	*p*
Intercept	-	-	*Not removed*	- 0.4024	- 2.539	.0114
Valence	-	-	*Not removed*	0.0557	2.172	.0303
Arousal	-	-	*Not removed*	0.0542	2.141	.0328
Valence : Arousal	-	-	*Not removed*	- 0.0086	- 2.082	.0378
Orthographic neighbours	0.1148	.7347	*1*	-	-	-
Concreteness: Valence: Arousal	0.5074	.4763	*2*	-	-	-
Concreteness : Valence	0.1226	.7262	*3*	-	-	-
Concreteness: Arousal	0.1499	.6986	*4*	-	-	-
Concreteness	0.082	.7746	*5*	-	–	-
Age of acquisition	0.3659	.5452	*6*	-	–	-
Frequency	0.7195	.3963	*7*	-	–	-
Length	2.8485	.09146	*8*	-	-	-

Table S3 summarizes the model-simplification procedure, including the goodness-of-fit tests and their results. Parameters were not removed when they were part of higher order interactions. The rightmost part of each table reports the effects of the included variables
