# Supplementary material for: Somatic and visceral effects of word valence, arousal and concreteness in a continuum lexical space
Source: Sci Rep. 2019 Dec 27;9:20254. doi: 10.1038/s41598-019-56382-2 (PMC6934768; doi:10.1038/s41598-019-56382-2)
Supplement: Supplementary file 4 — Table S4 [file 41598_2019_56382_MOESM4_ESM.pdf]

Somatic and visceral effects of word valence, arousal and concreteness in a continuum lexical space

Alessandra Vergallito [1,2+]*, Marco Alessandro Petilli [1+], Luigi Cattaneo [3,4], Marco Marelli [1,2]

1 Department of Psychology, University of Milano-Bicocca

2 Milan Center for Neuroscience (NeuroMi),

3 Center for Mind/Brain Sciences (CIMeC), University of Trento

4 Department of Neuroscience, Biomedicine and Movement, University of Verona, Verona, Italy

+ AV and MAP equally contributed to the manuscript

*Corresponding author:

alessandra.vergallito@unimib.it,

Department of Psychology, University of Milano Bicocca,

Piazza Ateneo Nuovo, 1, 20126 Milano, Italy.

Model selection on the levator labii superioris

Parameter removed	*Degree of Freedom*	*AIC*	*Log likelihood*	*p*
Full model	14	19310	*-9641.1*	-
Ortographical neighbours	13	19308	*-9641.2*	*.7347*
Concreteness: Valence: Arousal	12	19307	*-9641.3*	*.6044*
Concreteness : Valence	11	19305	*-9641.4*	*.7220*
Concreteness: Arousal	10	19303	*-9641.5*	*.7016*
Concreteness	9	19301	*-9641.5*	*.771*
Age of acquisition	8	19299	*-9641.7*	*.5463*
Frequency	7	19298	*-9642.0*	*.3930*
Length	6	19299	*-9643.5*	*.09343*
Valence: Arousal	5	19301	*-9645.6*	*.03662*

Table S4 summarizes degree of freedom, AIC, Log likelihood and p values of each model.

The first column ("parameter removed") indicates the removed parameter. Subsequent columns show the impact of the parameter removal in terms of model fit. Please note that models are in hierarchical level, starting from the full model and removing a parameter for each step.
