# Supplementary material for: Somatic and visceral effects of word valence, arousal and concreteness in a continuum lexical space
Source: Sci Rep. 2019 Dec 27;9:20254. doi: 10.1038/s41598-019-56382-2 (PMC6934768; doi:10.1038/s41598-019-56382-2)
Supplement: Supplementary file 5 — Table S5 [file 41598_2019_56382_MOESM5_ESM.pdf]

***Somatic and visceral effects of word valence, arousal and concreteness in a continuum lexical space***

Alessandra Vergallito [1,2+]*, Marco Alessandro Petilli [1+], Luigi Cattaneo [3,4], Marco Marelli [1,2]

1 Department of Psychology, University of Milano-Bicocca

2 Milan Center for Neuroscience (NeuroMi),

3 Center for Mind/Brain Sciences (CIMeC), University of Trento

4 Department of Neuroscience, Biomedicine and Movement, University of Verona, Verona, Italy

+ AV and MAP equally contributed to the manuscript

*Corresponding author:

alessandra.vergallito@unimib.it,

Department of Psychology, University of Milano Bicocca,

Piazza Ateneo Nuovo, 1, 20126 Milano, Italy.

*Model selection on the zygomaticus major*

| *Parameter* | χ2 | *p* | *Removal order* | *Estimate* | *t-value* | *p* |
|---|---|---|---|---|---|---|
| *Intercept* | - | | *Not removed* | *-0.035282* | *-3.045* | *.0039* |
| *Orthographic neighbours* | - | | *Not removed* | -0.003871 | *-2.136* | *.03313* |
| *Length* | 0.0003 | .9854 | *1* | - | - | - |
| *Frequency* | 0.0355 | .8505 | *2* | - | - | - |
| *Concreteness : Valence : Arousal* | .2357 | .6273 | *3* | - | - | - |
| *Age of Acquisition* | .3525 | .5527 | *4* | - | - | - |
| *Concreteness : Arousal* | 1.7929 | .1806 | *5* | - | - | - |
| *Concreteness: Valence* | 1.9453 | .1631 | *6* | - | - | - |
| *Concreteness* | 0.5051 | .4773 | *7* | - | - | - |
| *Valence: Arousal* | 2.9706 | .0848 | *8* | - | - | - |
| *Arousal* | 0.066 | .7973 | *9* | - | - | - |
| *Valence* | 0.1881 | .6645 | *10* | - | - | - |

*Table S5 summarizes the model-simplification procedure, including the goodness-of-fit tests and their results. Parameters were not removed when they were part of higher order interactions. The rightmost part of each table reports the effects of the included variables*
