# Supplementary material for: Somatic and visceral effects of word valence, arousal and concreteness in a continuum lexical space
Source: Sci Rep. 2019 Dec 27;9:20254. doi: 10.1038/s41598-019-56382-2 (PMC6934768; doi:10.1038/s41598-019-56382-2)
Supplement: Supplementary file 7 — Table S7 [file 41598_2019_56382_MOESM7_ESM.pdf]

Somatic and visceral effects of word valence, arousal and concreteness in a continuum lexical space

Alessandra Vergallito [1,2+]*, Marco Alessandro Petilli [1+], Luigi Cattaneo [3,4], Marco Marelli [1,2]

1 Department of Psychology, University of Milano-Bicocca

2 Milan Center for Neuroscience (NeuroMi),

3 Center for Mind/Brain Sciences (CIMeC), University of Trento

4 Department of Neuroscience, Biomedicine and Movement, University of Verona, Verona, Italy

+ AV and MAP equally contributed to the manuscript

*Corresponding author:

alessandra.vergallito@unimib.it,

Department of Psychology, University of Milano Bicocca,

Piazza Ateneo Nuovo, 1, 20126 Milano, Italy.

Model selection on the heart rate

Parameter	χ2	*p*	*Removal order*	*Estimate*	*t-value*	*p*
Intercept	-	-	*Not removed*	1.802	2.023	.0437
Concreteness	-	-	*Not removed*	-0.2701	-1.935	.0536
Valence	-	-	*Not removed*	-0.3155	-2.236	.0258
Arousal	-	-	*Not removed*	-0.2755	-1.925	.0549
Concreteness: Valence	-	-	*Not removed*	0.0464	2.109	.0355
Concreteness : Arousal	-	-	*Not removed*	0.0416	1.833	.0675
Valence : Arousal	-	-	*Not removed*	0.0492	2.167	.0307
Concreteness : Valence : Arousal	-	-	*Not removed*	-0.0075	-2.080	.0381
Orthographic neighbours	*0.0049*	*.9443*	*1*	-	-	-
Frequency	*0.559*	*.4547*	*2*	-	-	-
Age of acquisition	*2.6654*	*.1026*	*3*	-	-	-
Length	*2.7028*	*.1002*	*4*	-	-	-

Table S7 reports the results of the mixed-effect analysis on heart rate change. The table summarizes the model-simplification procedure, including the goodness-of-fit tests and their results. Parameters were not removed when they were part of higher order interactions. The rightmost part of each table reports the effects of the included variables
