# Supplementary material for: Somatic and visceral effects of word valence, arousal and concreteness in a continuum lexical space
Source: Sci Rep. 2019 Dec 27;9:20254. doi: 10.1038/s41598-019-56382-2 (PMC6934768; doi:10.1038/s41598-019-56382-2)
Supplement: Supplementary file 9 — Readme [file 41598_2019_56382_MOESM9_ESM.pdf]

Somatic and visceral effects of word valence, arousal and concreteness in a continuum lexical space

Alessandra Vergallito [1,2+*], Marco Alessandro Petilli [1+], Luigi Cattaneo [3,4], Marco Marelli [1,2]

1 Department of Psychology, University of Milano-Bicocca

2 Milan Center for Neuroscience (NeuroMi),

3 Center for Mind/Brain Sciences (CIMeC), University of Trento

4 Department of Neuroscience, Biomedicine and Movement, University of Verona, Verona, Italy

+ AV and MAP equally contributed to the manuscript

*Corresponding author:

alessandra.vergallito@unimib.it,

Department of Psychology, University of Milano Bicocca,

Piazza Ateneo Nuovo, 1, 20126 Milano, Italy.

#The dataset includes electromyographic activity from three facial muscles (left corrugator supercilii,cor, zygomaticus major, zig, and levator labii superioris, lab) and changes in the heart rate in response to 500 Italian word auditory stimuli from 20 participants. Word stimuli were extracted from the Italian adaptation of the Affective norms for English words (ANEW) by Montefinese et al. (2014). "NA" corresponds to discarded trials due to the presence of artifactual activity in the facial EMG signal.

FILE: Database.xlsx

Ita_Word = stimulus in Italian

Eng_Word = English translation of the stimulus

ID = participant identification number

cor = left corrugator supercilii EMG activity expressed in z-score

lab = levator labii superioris EMG activity expressed in z-score

zig = zygomaticus major EMG activity expressed in z-score

hr = heart rate change expressed in z-score
